# Two Crinivirus-Conserved Small Proteins, P5 and P9, Are Indispensable for Efficient *Lettuce infectious yellows virus* Infectivity in Plants

**DOI:** 10.3390/v10090459

**Published:** 2018-08-28

**Authors:** Wenjie Qiao, Erin L. Helpio, Bryce W. Falk

**Affiliations:** Department of Plant Pathology, University of California, Davis, CA 95616, USA; qwenjie@ucdavis.edu (W.Q.); elhelpio@ucdavis.edu (E.L.H.)

**Keywords:** *Lettuce infectious yellows virus*, P5, P9, small ORFs, virus infectivity, ER stress

## Abstract

Genomic analysis of *Lettuce infectious yellows virus* (LIYV) has revealed two short open reading frames (ORFs) on LIYV RNA2, that are predicted to encode a 5-kDa (P5) and a 9-kDa (P9) protein. The P5 ORF is part of the conserved quintuple gene block in the family *Closteroviridae*, while P9 orthologs are found in all *Criniviruses*. In this study, the expression of LIYV P5 and P9 proteins was confirmed; P5 is further characterized as an endoplasmic reticulum (ER)-localized integral transmembrane protein and P9 is a soluble protein. The knockout LIYV mutants presented reduced symptom severity and virus accumulation in *Nicotiana benthamiana* or lettuce plants, indicating their importance in efficient virus infection. The P5 mutant was successfully complemented by a dislocated P5 in the LIYV genome. The structural regions of P5 were tested and all were found to be required for the appropriate functions of P5. In addition, P5, as well as its ortholog P6, encoded by *Citrus tristeza virus* (CTV) and another ER-localized protein encoded by LIYV RNA1, were found to cause cell death when expressed in *N. benthamiana* plants from a TMV vector, and induce ER stress and the unfolded protein response (UPR).

## 1. Introduction

RNA viruses have evolved to compress maximal coding and regulatory information into minimal sequence space. Small open reading frames (ORFs) that encode peptides of less than 100 amino acids, a strategy often employed by viruses to minimize the size of their genome, still perform essential roles in virus infection [1]. For example, the 6-kDa viral protein 6K2 of *Turnip mosaic virus* (TuMV, *Potyvirus*) is a membrane-associated protein that induces the formation of endoplasmic reticulum (ER)-derived vesicles for viral genome amplification, and mediates their export from the ER for virus systemic infection [2,3]. Another 6-kDa potyviral protein, 6K1, is also required for viral replication and targets the viral replication complex at the early stage of infection [4]. In another example, two consecutive small proteins of 7-kDa (P7A and P7B) encoded by *Melon necrotic spot virus* (MNSV) are involved in virus cell-to-cell movement, and P7B has shown to be a type II integral membrane protein that is essential for ER export, transport to the Golgi apparatus and finally to the plasmadesmata (PD) [5,6,7]. However, for many viruses, the very small ORFs/proteins are often overlooked due to their short sequences and uncertain significance. It is imperative to identify the coding regions and understand their functions to decipher how these small ORFs/proteins promote the infection cycle.

*Lettuce infectious yellows virus* (LIYV) is a positive-sense RNA virus belonging to the genus *Crinivirus* in the family *Closteroviridae*. It has a bipartite genome. RNA1 contains three ORFs encoding replication-associated proteins and alone is capable of replication: ORF1a is predicted to encode a protein with the conserved domains of papain-like cysteine proteinase (PRO), methyltransferase (MTR) and helicase (HEL); ORF1b codes the viral RNA-dependent RNA polymerase (RdRp); ORF2 encodes a 34-kDa protein, P34, that is required for LIYV RNA2 replication [8,9]. RNA2 is predicted to encode seven proteins. For five of them, the protein products have been detected and partially characterized, including four virion proteins, CP (major coat protein), CPm (minor coat protein), Hsp70h, P59, and P26, which induces a LIYV-specific cytopathology, plasmalemma deposits [10,11,12,13]. The remaining LIYV RNA 2 ORF1 and ORF4 are predicted to encode a ~5-kDa (39 amino acids) and a ~9-kDa (80 amino acids) protein, P5 and P9, respectively. The P5 ORF is part of a gene block called the ‘hallmark *Closterovirus* gene array’ that is conserved among all members in the family *Closteroviridae*, and P5 is predicted to be highly hydrophobic with a transmembrane helix [14]. *Beet yellows virus* (BYV, *Closterovirus*)-encoded P6, an ortholog of P5, has been predicted to be a type III integral membrane protein localized to the ER that plays a role in BYV cell-to-cell movement [15,16]. The P9 ORF, on the other hand, is located in the central region of the LIYV RNA2, and similarly positioned P9 ORFs encoding for proteins similar in size are found in all the members of the genus *Crinivirus* sequenced so far [17]. However, whether or not these small ORFs encode for proteins during infection, and if so, what their roles are in infection are unknown. In this study, we addressed these questions. We demonstrated that the P5 and P9 proteins are indeed produced in LIYV-infected plants, and are essential for efficient viral infectivity. P5 was further indicated as an ER-localized integral membrane protein that was capable of inducing ER stress, and activating the unfolded protein response (UPR) and cell death when expressed in *N. benthamiana* plants from a TMV vector.

## 2. Materials and Methods

### 2.1. Secondary Structure and Transmembrane Domain Prediction

Secondary structures of P5 and P9 were predicted using the I-TASSER (https://zhanglab.ccmb.med.umich.edu/I-TASSER/) and Jpred 4 (http://www.compbio.dundee.ac.uk/jpred/) servers [18,19]. The transmembrane domain prediction of P5 was done using the following algorithms available online: SOSUI (http://harrier.nagahama-i-bio.ac.jp/sosui/sosui_submit.html) [20], DAS (https://tmdas.bioinfo.se/DAS/index.html) [21], TMHMM (http://www.cbs.dtu.dk/services/TMHMM/) [22], CCTOP (http://cctop.enzim.ttk.mta.hu) [23], Phobius (http://phobius.sbc.su.se) [24], and TOPCONS (http://topcons.cbr.su.se) [25].

### 2.2. Molecular Cloning

All plasmid constructs used in this work were generated using In-Fusion HD Cloning (Clontech, Mountain View, CA, USA) following manufacturer’s instructions. To generate P5- and P9-GFP fusion constructs for transient expression in *Nicotiana benthamiana* epidermal cells, P5 and P9 sequences cloned from the LIYV RNA2 infectious clone were assembled into the pEAQ-HT vector [26]. The GFP ORF was then ligated into their N- or C-termini as an in-frame coding sequence. Constructs including the knockout mutants (P5X, P9X) and the deletion mutants (P5Δ, P9Δ) were generated from the LIYV RNA2 infectious clone [27], the P5X-GFP and P9X-GFP constructs were modified from the GFP-tagged LIYV vector [28], and the P5 truncation mutants were derived from pEAQHT-P5GFP (P5_O_Δ:GFP, P5_TMS_Δ:GFP, P5_IN_Δ:GFP) or from the LIYV RNA2 infectious clone (P5_O_Δ, P5_TMS_Δ, P5_IN_Δ). The mutagenesis was introduced into the existing constructs through In-Fusion Cloning using primers to incorporate the mutations outside the unwanted region and then re-circularize into desired clones. The P5X-P5 construct was generated by replacing the GFP ORF of the P5X-GFP construct with the *P5* gene. The plasmid backbones or insertion sequences were amplified by PCR using CloneAmp HiFi PCR Premix (Clontech).

For heterologous expression of LIYV ORFs from the pTRBO vector [29], the pTRBO plasmid was linearized with PacI and NotI and ligated with the cloned LIYV ORFs separately using In-Fusion HD Cloning.

### 2.3. Transient Expression, Virus Inoculation and Fluorescence Detection

Four- to six-leaf stage Hc-Pro transgenic *N. benthamiana* plants and *Agrobacterium tumefaciens* strain GV3101 were used for transient expression analysis and virus inoculation as described before [27,28]. The subcellular localization of the GFP fusion proteins within agroinfiltrated *N. benthamiana* epidermal cells was examined using a Leica TCS SP2 inverted confocal microscope (Leica Microsystems, Wetzlar, Germany) under a 63× water immersion objective, with excitation/emission filter wavelengths: 488/497 to 520 nm for GFP and 561/585 to 615 nm for RFP. GFP expression in *N. benthamiana* plant leaves was visualized with a long-wavelength UV light and photographed using a Canon EOS 600D digital camera (Canon, Tokyo, Japan).

To test the effects of the LIYV mutants on lettuce plants (*Lactuca sativa* L.), virions were purified from agroinfected *N. benthamiana* plants, acquired by whiteflies through membrane-feeding, and transmitted to two- to three-leaf stage young lettuce plants as previously described [28,30].

### 2.4. Subcellular Fractionation and Chemical Treatment

Subcellular fractionation was performed as described by Donald, et al. [31]. *N. benthamiana* leaf tissues (1 g) were homogenized in 4 mL lysis buffer I (400 mM sucrose, 100 mM Tris, 10 mM KCl, 5 mM MgCl_2_, 10% glycerol, 10 mM β-mercaptoethanol, 1 mM phenylmethylsulfonyl (PMSF), pH 7.5). The homogenate was filtered through Miracloth (Sigma-Aldrich, Darmstadt, Germany) and the trapped debris was washed several times with lysis buffer containing 2% Triton X-100 (Calbiochem, Los Angeles, CA, USA) to generate the cell wall fraction (CW). The filtrate was centrifuged at 1000× *g* for 10 min to yield a low-speed pellet fraction (P1) and the supernatant was centrifuged at 30,000× *g* for 30 min to produce the soluble (S30) and the crude membrane (P30) fractions.

For chemical treatment, the P30 membrane pellets were resuspended in original lysis buffer containing 1% Triton X-100, 100 mM Na_2_CO_3_ (pH 11) or 4 M urea, and incubated on ice for 30 min [15]. The samples were precipitated again at 30,000× *g* to separate the P30 and S30 fractions, and the resulting P30 pellet was resuspended in lysis buffer in a volume equal to that of the original sample.

### 2.5. Sucrose Gradient Centrifugation

*N. benthamiana* leaf tissues were ground in lysis buffer II (20 mM HEPES (pH 6.8), 150 mM potassium acetate, 250 mM mannitol, 5 mM MgCl_2_). P30 prepared as described above was loaded on top of 20% to 60% linear sucrose gradients containing lysis buffer [15]. Gradients were centrifuged for 4 h at 100,000× *g* in a Beckman SW40 rotor (Beckman Coulter, Brea, CA, USA) at 4 °C, and 21 fractions of 0.6 mL each were collected from top to bottom using an ISCO model 640 gradient fractionator while monitoring absorption at 280 nm with an ISCO model UA-5 absorbance detector (ISCO, Lincoln, NE, USA). Aliquots of these fractions were analyzed by immunoblotting.

### 2.6. RT-PCR and RT-qPCR

RT-PCR and RT-qPCR were performed as described before [28]. The P5 and P9 mutations were checked by RT-PCR using primer sets LIYV-P5_F (5′-ATAGCAGTAGCTGTCGCAAACC-3′)/LIYV-P5_R (5′-ACTTTTTATTGATAAAATACTTATTACAAATT-3′) and LIYV-P9_F (5′-CATCACGGAAGATAGGAGTGAG-3′)/LIYV-P9_R (5′-TGTCAGCTTCATGATCCCTG-3′) which amplify sequences flanking the *P5* and *P9* genes, respectively. The relative LIYV RNA accumulation level in *N. benthamiana* plants was determined by RT-qPCR as mentioned by Qiao, et al. [28], while for tests with lettuce plants, the lettuce TIP41-like protein (TIP41) transcripts quantified with TIP41_F: 5′-GAGAGATTTGCTGGAGGGAAACTA-3′, TIP41_R: 5′-CCTTTGACTGATGATGTTTGGA-3′ and TIP41_Probe: 5′-TCCGCATTCATGGCTGGGAGAT-3′ were used as an internal control. To quantify the transcript levels of the stress-related *N. benthamiana* genes, qPCR was performed employing a SYBR Green supermix with gene specific primers, and the amplified 18S rRNA was used as an internal control. The primer sequences used are listed in Table S1. qPCR was performed and analyzed with a CFX96 real-time PCR detection system (Bio-Rad, Hercules, CA, USA).

### 2.7. Immunoblot Analysis

The 5-kDa and 9-kDa small proteins were separated on 13% Tris-Tricine polyacrylamide gels. Proteins of molecular weight larger than 20-kDa were analyzed with 12% Tris-Glycine polyacrylamide gels, and then processed for immunoblotting, following the protocols described before [12,28,32]. Primary antibodies used included anti-GFP (Thermo, Waltham, MA, USA), anti-LIYV CP, anti-LIYV P26, anti-LIYV P5, anti-LIYV P9, and anti-BiP (Agrisera, Vännäs, Sweden) protein polyclonal antibodies produced in rabbit. The secondary antibody was horseradish peroxidase-conjugated goat anti-rabbit IgG.

## 3. Results

### 3.1. P5 Is an Integral Membrane Protein and P9 Is a Soluble Protein

**Figure 1:**
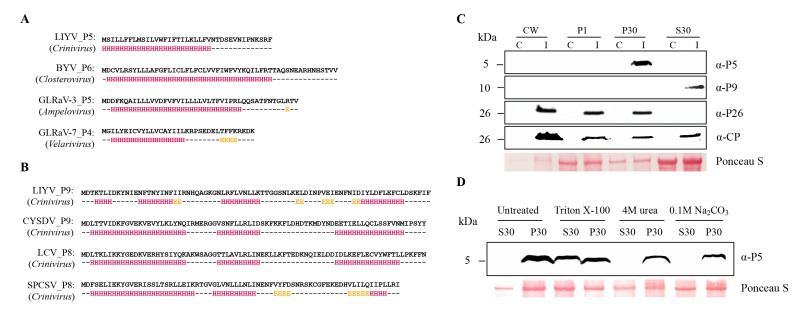
Detection and characterization of *Lettuce infectious yellows virus* (LIYV) P5 and P9 proteins. Predicted secondary structures of *Closterovirus* P5 orthologs (**A**) and *Crinivirus* P9 orthologs (**B**), computed using JPred 4 (http://www.compbio.dundee.ac.uk/jpred/). Residue positions predicted to participate in alpha helix formation are highlighted in red, positions predicted to participate in beta sheet structures are highlighted in yellow. Amino acid sequences used for secondary structure predictions were translated from sequences obtained from NCBI: LIYV RNA2 (NC_003618), *Beet yellows virus* (BYV, NC_001598), *Grapevine leafroll-associated virus*-3 (GLRaV-3, GQ352633), *Grapevine leafroll-associated virus*-7 (GLRaV-7, NC_016436), *Cucurbit yellow stunting disorder virus* (CYSDV RNA2, NC_004810), *Lettuce chlorosis virus* (LCV RNA2, NC_012910), *Sweet potato chlorotic stunt virus* (SPCSV RNA2, KC888962). (**C**) Cellular fractionation of P5 and P9 detected from LIYV-infected *Nicotiana benthamiana* plants. Four fractions, cell wall (CW), 1000× *g* pellet (P1), 30,000× *g* pellet (P30), and supernatant (S30), were analyzed by immunoblots using antibodies against LIYV P5 and P9. The previously studied LIYV P26 and CP fractionations were used as operating controls. I = LIYV-infected sample; C = buffer-inoculated control. (**D**) P30 fractions of P5 were incubated with 1% Triton X-100, 100 mM Na_2_CO_3_ (pH 11) or 4 M urea, followed by centrifugation and immunoblotting using P5 antibody. The Ponceau S stained rubisco large subunit serves as a loading control.

The LIYV P5 and P9 amino acid sequences as well as their orthologs were analyzed using the protein structure prediction programs I-TASSER and Jpred 4. These consistently predicted that P5 orthologs are membrane proteins with a single α-helical transmembrane domain (Figure 1A, also see Table 1). This comparison is among *Closteroviruses* of different genera but still suggests similarity of these small proteins. The *Crinivirus*-specific P9 orthologs are predicted to be soluble proteins featuring three conserved short alpha helix regions (Figure 1B). Despite the lack of amino acid similarity, the *Crinivirus* orthologs show similarity in their predicted secondary structures.

To determine if these small proteins could be detected from LIYV-infected plants, and if so, to assess their subcellular association, protein extracts derived from the leaf tissues of the LIYV-infected *N. benthamiana* plants were analyzed through subcellular fractionation. The protein extracts were subjected to differential centrifugation to yield the cell wall fraction (CW), 1000× *g* pellet fraction (P1), 30,000× *g* pellet fraction (P30) and a supernatant fraction (S30). Each fraction was analyzed by SDS-PAGE and immunoblotting using LIYV P5 and P9 specific antibodies raised from synthetic peptide antigens. P5 was found enriched in the P30 fraction, representing a crude membrane fraction [31], while P9 was detected in the S30 fraction enriched for soluble proteins, suggesting that P5 is a membrane-associated protein and P9 is a soluble protein, which is consistent with the predicted protein features (Figure 1C). The fractionation patterns of LIYV CP and P26 were also analyzed as controls and the results were consistent with previous findings (Figure 1C) [12,13].

The membrane association of P5 was further characterized by solubilizing P5 from the P30 fraction by the treatment with the non-ionic detergent Triton X-100 (Figure 1D). The treatment of the P30 fractions of P5 with 0.1 M Na_2_CO_3_ (pH 11.0) and 4 M urea was employed to assess whether P5 is a peripheral or integral membrane protein [31,33]. The result that neither released the P5 protein into the soluble fraction further defined P5 as an integral membrane protein (Figure 1D).

### 3.2. P5 is Localized to the ER

**Figure 2:**
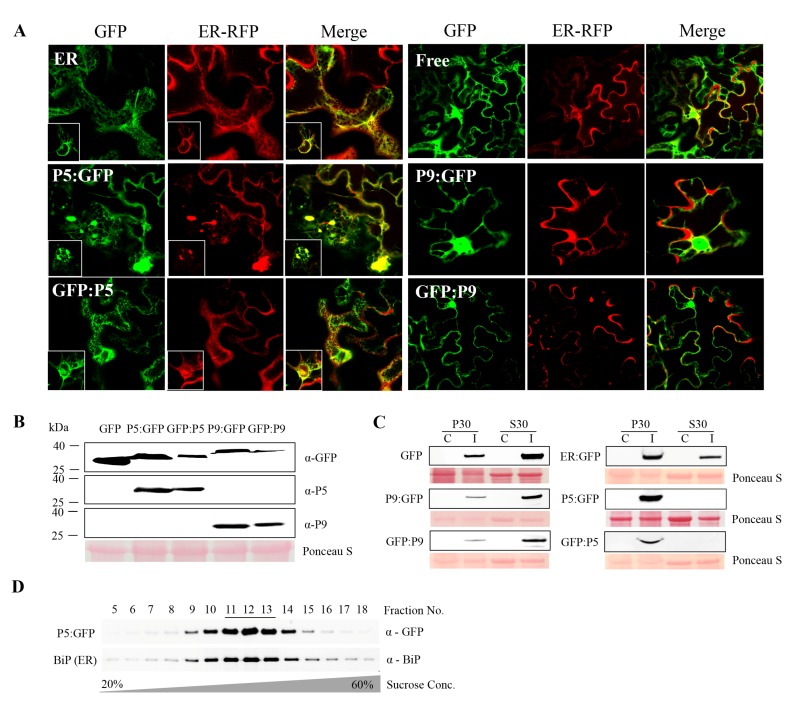
The endoplasmic reticulum (ER) association of LIYV P5. (**A**) Confocal microscopy imaging of leaf epidermal cells of *Nicotiana benthamiana* plants co-infiltrated with P5- or P9-GFP fusion proteins and ER-localized RFP constructs at two days post-infiltration (dpi) obtained under a 63× water immersion objective. Free GFP and ER-localized GFP were used as controls. The white square shows the nucleus of the photographed cell. (**B**) Fusion protein expression detected by immunoblotting using GFP, LIYV P5 and P9 specific antibodies. Free GFP was used as a control. (**C**) Cellular fractionation of P5 and P9 GFP fusion proteins. The 30,000× *g* pellet (P30) and supernatant (S30) fractions were analyzed by immunoblots using anti-GFP antibody. Free GFP and ER-localized GFP were examined as controls. The Ponceau S stained rubisco large subunit serves as a loading control. (**D**) Immunoblot analysis of the P30 fraction of P5:GFP fusion protein following separation in 20% to 60% sucrose gradients. Fractions from top to bottom (5 to 18) were analyzed by immunoblots using anti-GFP and anti-BiP antibodies to detect P5:GFP and BiP (ER-resident marker protein).

Next, we wished to visualize the localization of P5 and P9 within cells. For this, GFP was separately fused to their N- (GFP:P5, GFP:P9) and C-termini (P5:GFP, P9:GFP), respectively, and fusions were ligated into the binary vector pEAQ-HT. Recombinant proteins were transiently expressed in the epidermal cells of fully expanded *N. benthamiana* leaves by agroinfiltration and leaves were examined using confocal laser scanning microscopy. Both P5:GFP and GFP:P5 were found co-localized with the cortical ER marker RFP:HDEL [34], while the former induced obvious ER rearrangement, forming aggregate structures. In contrast, P9 constructs showed the typical nuclear and cytoplasmic distribution patterns similar to the free GFP control (Figure 2A). The expression of these fusion proteins was confirmed by immunoblotting with anti-GFP, P5, and P9 antibodies (Figure 2B). The transiently expressed P5 and P9 fusion proteins were partitioned to the same subcellular fractions as those produced upon virus infection. P5 fusion proteins were mainly detected in the P30 pellet fraction, while P9 fusion proteins were mostly found in the soluble S30 fraction (Figure 2C).

The ER localization of P5 was then confirmed by using sucrose density gradient centrifugation to fractionate P5:GFP-containing membranes derived from transiently expressed P5:GFP in *N. benthamiana* leaves and comparing them with ER marker proteins. Immunoblot analysis of the gradient fractions using P5-specific antibody revealed the peak of P5:GFP accumulation in fractions 11 to 13 (Figure 2D). Indeed, probing the same gradient fractions with the antibody of a resident ER protein, BiP, demonstrated co-fractionation of BiP and P5, thus providing additional evidence for the ER localization of P5 (Figure 2D).

### 3.3. The Roles of P5 and P9 in LIYV Infection Assessed by Mutational Analysis

**Figure 3:**
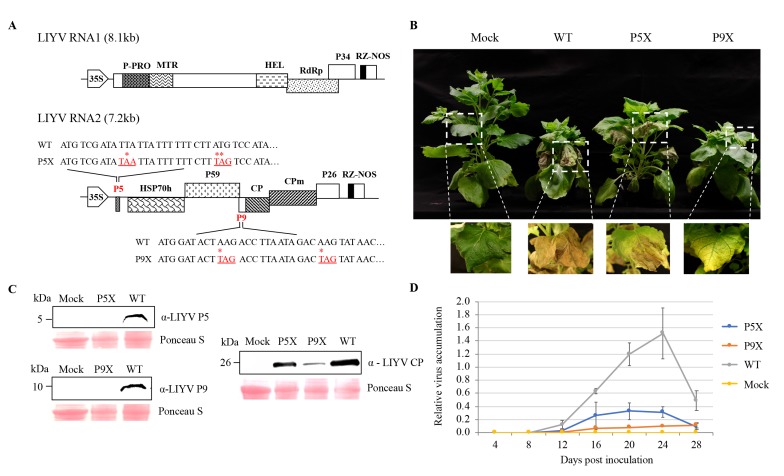
Mutational analysis of P5 and P9 functions in LIYV infection of *Nicotiana benthamiana* plants. (**A**) Schematic representation of the genomic organization of LIYV cDNA infectious clones. LIYV P5 and P9 knockout mutants (P5X, P9X) were constructed by introducing two in-frame stop codons near the 5′ termini of the P5 and P9 ORFs of the LIYV wild-type (WT). The nucleotides replaced are marked with an asterisk (*) and addressed in red. NOS, nopaline synthase terminator; 35S, 35S promoter; the boxes indicate open reading frames (ORF) and their corresponding translation products. (**B**) *N. benthamiana* plants infected by LIYV WT, P5X and P9X photographed at four weeks post inoculation (wpi). Mock indicates buffer-inoculated control. (**C**) LIYV P5, P9 and CP accumulation in upper non-inoculated leaves analyzed by immunoblotting. The Ponceau S stained rubisco large subunit serves as a loading control. (**D**) LIYV RNA1 accumulation in upper non-inoculated leaves of LIYV WT, P5X and P9X infected plants quantified by RT-qPCR at time points indicated. Error bars indicate standard errors from at least three biological replicates.

To investigate the roles of P5 and P9 in LIYV infections, mutants were generated by introducing two in-frame stop codons close to the 5′-termini of their ORFs, respectively, in the wild-type LIYV infectious clone (WT), hereafter referred to as P5X and P9X (Figure 3A). The WT and the mutant clones were delivered by agroinfiltration to Hc-Pro transgenic *N. benthamiana* plants, which overexpress the P1/HC-Pro silencing suppressor of *Turnip mosaic virus*, which could thus enhance LIYV accumulation and symptom development [27]. At around 2 wpi, plants inoculated with WT and P9X started to show typical LIYV symptoms: stunting and interveinal chlorosis, while the symptom development on P5X-infected plants was delayed by about one week. In addition, symptomatic leaves of WT and P5X-infected *N. benthamiana* plants showed necrosis, whereas P9X-infected plants showed only stunting and interveinal yellowing symptoms at the late stages of infection (Figure 3B). Next, P5 and P9 ORF deletion mutants (P5Δ, P9Δ) were constructed, and these gave similar phenotypes corresponding to those seen for P5X and P9X (Figure S1). Mutation retention of P5 and P9 mutants was confirmed by two methods. First, we used RT-PCR with RNA extracted from the upper non-inoculated leaves at 4 wpi using primers flanking the mutation sites and sequencing. No mutation reversion was detected (Figure S1). Second, the lack of P5 and P9 protein expression was verified by immunoblotting with anti-P5 and P9 antibodies (Figure 3C).

All P5 and P9 mutants were still able to systemically infect and cause symptoms in plants, thus neither gene/protein was absolutely required for LIYV infection, but knockouts of either affected phenotype. Therefore, we compared LIYV RNA and coat protein accumulation levels in *N. benthamiana* plants inoculated with WT and the P5X, P9X mutants. Upper non-inoculated leaves were collected every 4 dpi to quantify LIYV RNA accumulation by RT-qPCR (Figure 3D). The LIYV coat protein accumulation in upper leaves was also quantified by immunoblotting and confirmed the RT-qPCR results. Both P5X and P9X showed reduced efficiency of systemic infection as compared to LIYV WT (Figure 3C). The reduced accumulation compared to the WT may partially explain their delayed and/or reduced symptom development. It is interesting to note that P9X-infected plants showed stunting in *N. benthamiana* plants similar to that seen for WT, but P9X plants had the lowest LIYV titer. The systemically reduced virus accumulation levels of both mutants were also confirmed by visualizing directly under UV light utilizing a GFP-tagged LIYV vector (WT-GFP) we developed before [28]. The GFP intensity reflected virus accumulation levels (Figure S2).

**Figure 4:**
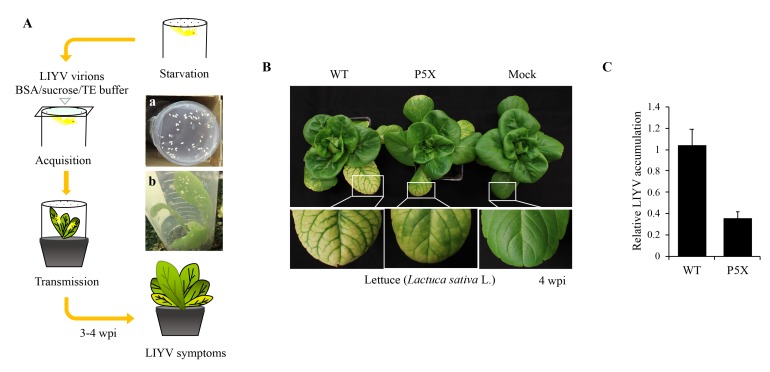
Mutational analysis of P5 function in LIYV infection of lettuce (*Lactuca sativa* L.) plants. (**A**) Schematic representation of LIYV virions purified from systemically infected *Nicotiana benthamiana* plants and transmitted by whiteflies (*Bemisia tabaci*) to lettuces. *B. tabaci* were placed in the feeding tube with one side covered with single layer of Parafilm and were starved for 4 h. Then, 10 µL artificial sucrose diet containing LIYV particles was added on the top of the Parafilm, which was covered with a microscope cover slip to spread the solution, and whiteflies were fed for 6–10 h (a). The viruliferous *B. tabaci* were transferred onto young lettuce plants for virus inoculation (b). Whiteflies were eliminated with insecticide after a three-day inoculation access period. Inoculated lettuce plants were sprayed with insecticide and kept in a greenhouse for 3–4 weeks and assessed by symptoms. (**B**) Phenotypes of lettuce plants inoculated with *B. tabaci* fed with buffer (Mock), LIYV WT and P5X virions at 4 wpi. (**C**) Quantification of LIYV RNA1 accumulation in LIYV WT and P5X infected lettuce plants by RT-qPCR. The TIP41 transcript level of lettuces was used as an internal control. Error bars denote standard errors from at least three biological replicates.

The above results were obtained using agroinoculated *N. benthamiana* plants. Therefore, we next attempted to test the effects of the P5X and P9X mutations on LIYV infected lettuce (*Lactuca sativa* L.) plants. We used the whitely vector, *Bemisia tabaci*, and membrane feeding assays for these experiments as LIYV agroinoculation is only practical for *N. benthamiana* plants and *B. tabaci* whiteflies do not efficiently feed on *N. benthamiana* plants to acquire LIYV directly from agroinoculated plants. Virions were recovered from all plants (Figure S1), although we were only able to recover low amounts from P9X-inculated plants. Thus, as illustrated in the schematic diagram (Figure 4A), nonviruliferous *B. tabaci* were allowed to feed on a sucrose diet containing purified LIYV virions for the WT or the P5X and P9X. After 6 h to 10 h, whiteflies were transferred to lettuce plants for virus inoculation. All plants inoculated by viruliferous *B. tabaci* carrying the WT and P5X virions became infected. Compared to the WT-infected lettuce plants that showed typical interveinal yellowing symptoms approximately three weeks post inoculation, the symptom development of the P5X mutant was delayed about 1–2 weeks along with a less severe yellowing phenotype (Figure 4B). Similar to results from *N. benthamiana* plants, P5X showed greatly reduced LIYV RNA accumulation (Figure 4C). We did not obtain the P9X-infected lettuce plants even after several trials. One possible explanation is that we always obtained a very low virion yield for P9X, even when we used a large amount of plant tissue for extraction.

### 3.4. P5X is Recovered by a Translocated P5 in the LIYV Genome

**Figure 5:**
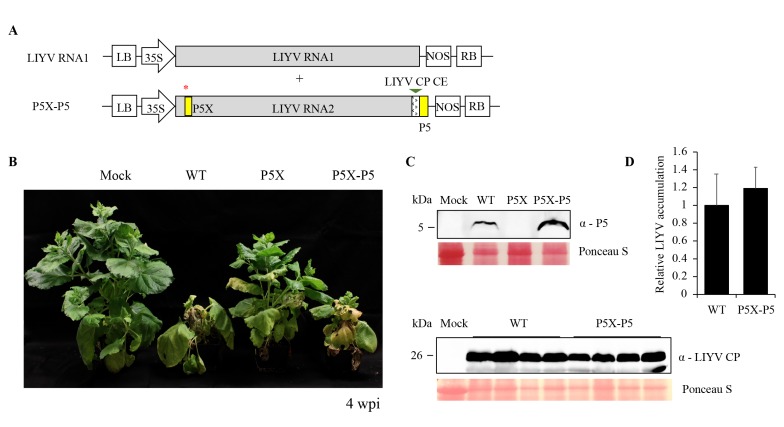
LIYV P5 mutant (P5X) complemented by a translocated P5 (P5X-P5) in the LIYV genome. (**A**) Schematic diagram of the genome organization of the P5X-P5 construct. The *P5* gene controlled by a LIYV CP controller element (CE) was inserted between the P26 ORF and 3′-nontranslated region (NTR) of LIYV RNA2. The red * indicates the mutated *P5* gene. (**B**) Phenotypes of LIYV WT, P5X and P5X-P5 agroinoculated *N. benthamiana* plants photographed at 4 wpi. (**C**) Immunoblot analysis of the LIYV P5 and CP expression in upper non-inoculated leaves of LIYV WT and P5X-P5 infected plants using LIYV P5- and CP-specific antibodies. Mock indicates buffer-inoculated control. The Ponceau S stained rubisco large subunit serves as a loading control. (**D**) Quantification of LIYV RNA1 accumulation in LIYV WT and P5X-P5 infected *N. benthamiana* plants by RT-qPCR. The PP2A transcript level of lettuces was used as an internal control. Error bars denote standard errors from at least three biological replicates.

In order to ensure that the properties seen for LIYV P5X were due to the introduced mutations, we used several complementation strategies in an attempt to restore the properties of LIYV P5X to be comparable to the WT. We first attempted to express the P5 protein *in trans* from a binary plant expression vector, or from a TMV vector, in leaves that were agroinoculated with LIYV P5X, but the locally expressed P5 protein failed to rescue the P5-defective phenotype, and P5-expressing TMV was also noticed to cause programmed cell death (Figure S3), which has been discussed further in Section 3.6. We then attempted to rescue P5X by expressing P5 *in cis*. We created a P5X-P5 construct, in which the GFP ORF of the P5X-GFP construct was replaced by the *P5* gene, thus incorporating a duplicated but translocated P5 ORF in the LIYV P5X genome (Figure 5A). After agroinoculation, the symptom development and phenotype of the P5X-P5 infected plants were observed to be indistinguishable to those of the LIYV WT infected plants (Figure 5B). As expected, comparable virus accumulation level was also detected by RT-qPCR and immunoblotting (Figure 5C,D). The P5 protein expression from the *cis*-complemented *P5* gene was confirmed by immunoblotting (Figure 5C). These data demonstrated that a translocated P5 in the LIYV genome could rescue the infection efficiency of the P5 knockout mutant, and that the properties seen for P5X were due to the specific, introduced mutations.

### 3.5. All Regions of P5 are Required for Efficient Virus Infection

**Figure 6:**
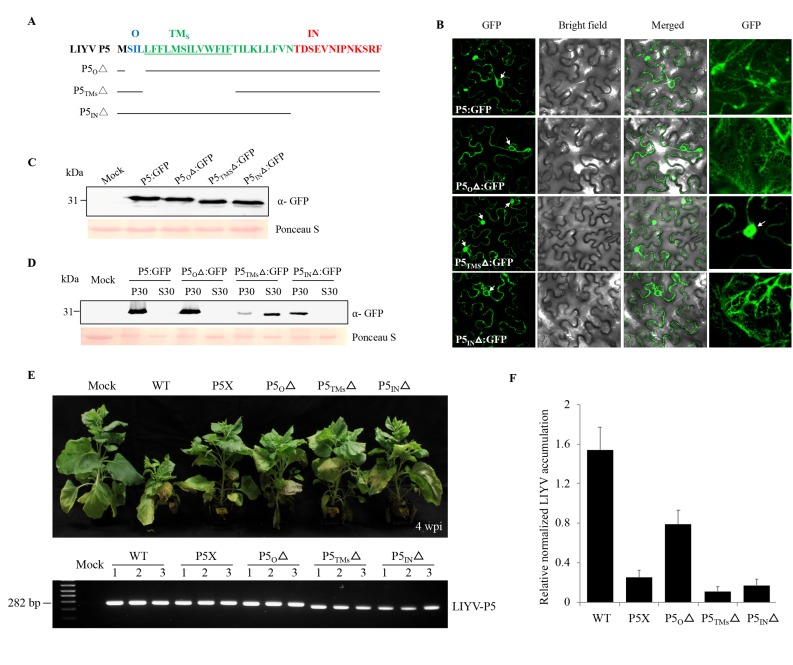
Evaluation of different regions of LIYV P5 contributing to viral infection. (**A**) The inner- (IN, red color), outer- (O, blue color) and trans- (TM, green color) membrane regions of P5 predicted by the SOSUI program. The minimal transmembrane (TM) region predicted is underlined. The deletion mutants of P5 were indicated by the solid lines with the names of the constructs listed at left. (**B**) Confocal imaging of P5-GFP fusion proteins containing P5 truncation mutations in *Nicotiana benthamiana* epidermal cells at 2 dpi obtained under a 63× water immersion objective. Arrows indicate cell nucleus. The right GFP column shows enlarged cellular structures of the ER and nucleus. (**C**) Fusion protein expression detected by immunoblotting using a GFP specific antibody. (**D**) Cellular fractionation of P5 mutant GFP fusion proteins. The 30,000× *g* pellet (P30) and supernatant (S30) fractions were analyzed by immunoblots using anti-GFP antibody. The Ponceau S stained rubisco large subunit serves as a loading control. (**E**) Phenotypes of LIYV WT and P5 mutants in agroinoculated *N. benthamiana* plants photographed at 4 wpi. P5 mutations were examined by RT-PCR using total RNAs extracted from upper non-inoculated leaves of these agroinoculated plants. LIYV P5 primer sets were used to amplify the sequence flanking the P5 ORF. Mock indicates buffer inoculated control. (**F**) Quantification of LIYV RNA1 accumulation in LIYV WT and P5 mutants infected *N. benthamiana* plants by RT-qPCR. The PP2A transcript level of lettuces was used as an internal control. Error bars denote standard errors from at least three biological replicates.

P5 is predicted to be a transmembrane protein and our biochemical and localization data showed it to associate with the ER. Therefore, to dissect LIYV P5 in further detail, transmembrane prediction programs were used to determine the sequences in LIYV P5 that may enable its insertion into, or association with membranes. Although the output varied slightly according to the algorithms used, one TM segment spanning residues 4–27 was identified (Table 1). A topological prediction model of P5 based on the SOSUI program was applied for the following analysis, which featured the inner- (27–39 aa), outer- (2–4 aa) and minimal trans-membrane (5–17 aa) domains of the P5 protein (Figure 6A). The significance of the predicted P5 domains was tested by truncation analysis; the three P5 mutants were first tested as GFP fusion proteins, designated P5_O_Δ:GFP, P5_TMS_Δ:GFP and P5_IN_Δ:GFP, and their subcellular localization was evaluated using confocal microscopy. As predicted, P5 lost its ER association only when the minimal transmembrane domain was deleted (Figure 6B,C). The membrane association was also confirmed by subcellular fraction. P5_TMS_Δ:GFP was detected mainly in the S30 fraction as a soluble protein, instead of precipitating in the P30 fraction (Figure 6D). The three P5 truncation mutations were also introduced into the LIYV WT and tested *in planta*. All mutants showed significantly reduced viral symptoms and accumulation levels (Figure 6E,F). Thus, although only the mutant P5_TMS_Δ showed localization and fractionation alterations, the *in planta* assays showed that all regions of P5 are required for efficient virus infection in *N. benthamiana* plants.

### 3.6. LIYV P5 Causes Cell Death When Expressed from TMV Vector

**Figure 7:**
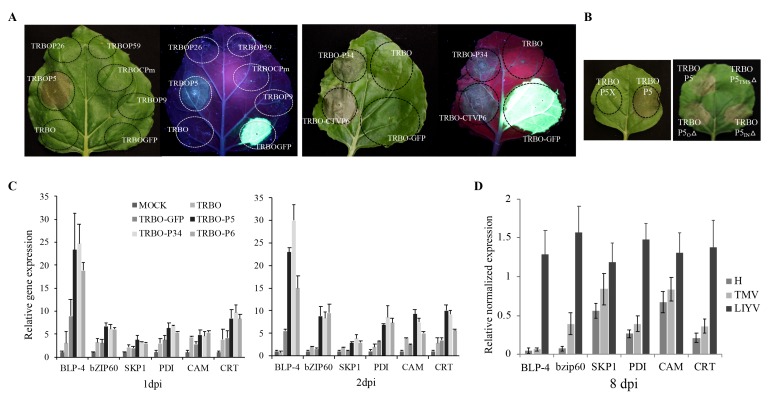
LIYV P5 causes cell death when expressed from a *Tobacco mosaic virus* (TMV) vector. (**A**) Expression of the LIYV P26, P59, CPm, P9, P34, P5 and *Citrus tristeza virus* (CTV) P6 from TRBO vector [29] on *Nicotiana benthamiana* leaves through agroinfiltration. Empty vector and vector expressing GFP were used as controls. GFP expression was visualized under UV light at 4 dpi. (**B**) Evaluation of different regions of LIYV P5 contributing to cell death on *N. benthamiana* leaves. (**C**) Quantification of BLP-4, bZIP60, SKP1, PDI, CRT, and CAM transcript abundance on *N. benthamiana* leaves inoculated with buffer (Mock), empty vector (TRBO), TRBO-GFP, TRBO-P5, TRBO-P34 and TRBO-P6 at 1 and 2 dpi. (**D**) Quantification of BLP-4, bZIP60, SKP1, PDI, CRT, and CAM transcript abundance on upper non-inoculated leaves of TMV and LIYV infected *N. benthamiana* plants at 8 dpi. The PP2A transcript level of lettuces was used as an internal control. Error bars denote standard errors from at least three biological replicates.

In the trial to complement the P5X with P5 protein expressed from a TMV-based binary vector (TRBO) [29], we noticed that the TRBOP5 construct caused hypersensitive response (HR)-like cell death on the agroinfiltrated leaf area at about 4 dpi (Figure 7A). We then tested the other six LIYV RNA2 proteins (Hsp70h, P59, CP, CPm, P9 and P26) in the TRBO vector. The empty vector and TRBOGFP were used as controls, but none induced the necrotic phenotype (Figure 7A). To further confirm that the cell death is caused by the expression of the P5 protein, two in-frame stop codons were introduced near the 5′-terminus of the P5 ORF as described for the LIYV P5X construct to generate TRBOP5X, and no cell death was observed (Figure 7B). Furthermore, P6, a P5 ortholog expressed from *Citrus tristeza virus* (CTV) and another LIYV ER-localized transmembrane protein, P34 encoded by LIYV RNA1, were also examined in the TRBO vector [9]. Both TRBOP34 and TRBOP6 caused the necrotic phenotype as expected, although the progress of cell death was slower within the TRBOP34 infiltrated area (~6 dpi) than for the TRBOP5 and TRBOP6 (~4 dpi) (Figure 7A). To further study whether the necrosis is caused by the transmembrane structure of LIYV P5, the truncation mutations of the different regions of P5 were introduced and tested. Only when the membrane-spanning region in the P5_TMS_Δ mutant was deleted was cell death eliminated (Figure 7B). Furthermore, the ER-localized triple gene block protein 3 (TGBp3) of *Potato virus* X (PVX, *Potexvirus*) and P11 encoded by *Garlic virus* X (GarVX, *Allexivirus*) were previously reported to induce ER stress and cell death when expressed from TMV, as well as the unfolded protein response (UPR), but not with another ER-localized PVX TGBp2 [35,36]. Therefore, we tested the transcript levels of several stress-related UPR indicator genes of *N. benthamiana*. The upregulation of basic-region leucine zipper 60 (bZIP60) and several endoplasmic reticulum (ER)-resident chaperones, including the ER luminal binding protein (BLP), SKP1, protein disulphide isomerase (PDI), calreticulin (CRT) and calmodulin (CAM) were detected with TRBOP5, TRBOP6 and TRBOP34 inoculated leaf tissues by 1 dpi, especially for BLP. These transcript levels were similar, or for some a little higher at 2 dpi (Figure 7C). Infection with LIYV in *N. benthamiana* plants also led to increased transcript levels of these host genes compared to plants infected by TMV (Figure 7D), which is reported to not induce the expression of UPR-related genes [35]. However, the TMV constructs used here did increase levels of some UPR genes compared to buffer inoculated controls, although much lower than that induced by LIYV infection and the TRBO vector plus viral proteins tested.

## 4. Discussion

Viruses in the family *Closteroviridae* possess the largest and most complex genomes (up to 20 kb) of all +ssRNA plant-infecting viruses, comprised of 9–13 ORFs encoding proteins for successful virus infection [37]. The two small ORFs coding for the P5 and P9 orthologs, either conserved among all members in the family *Closteroviridae* (P5) or in the genus *Crinivirus* (P9), are predicted based on nucleotide sequence and have been featured as part of their characteristic viral genomes. However, only one of the P5 orthologs encoded by the *Closterovirus* BYV, P6, has been shown to be produced in vivo during virus infection [15,16]. Since the ORFs are highly conserved, it seems that these small proteins are likely important for LIYV and other *Criniviruses*, but if so, what their roles are in virus infection are unknown. In this work, we demonstrated that P5 and P9 are expressed in LIYV-infected plants. Neither proved to be absolutely essential for LIYV infection, but knockouts of either led to debilitated LIYV infections.

LIYV P5 is an ER-localizing protein. Positive-strand RNA viruses are known to recruit endomembranes for the formation of spherules and vesicles, which provide protective environments and enlarged surfaces for not only viral genome replication, translation and particle assembly, but also for intracellular and cell-to-cell movement [38,39,40]. The movement proteins of several plant viruses were found in tight association with the ER and behaved as integral membrane proteins to support viral movement, as exemplified by BYV P6 (a LIYV P5 ortholog), TuMV 6K2 and MNSV P7B [3,6,7,15], which share a similar protein topology with LIYV P5. The mutagenesis analysis of LIYV P5 showed that its knockout mutant was still capable of systemic infection, but with delayed symptom development and reduced viral accumulation. However, the exact role of P5 involved in the occurrence of this phenotype remains unknown.

P5 was also found to induce severe ER stress due to its transmembrane structure, as shown by the ER aggregation visualized with P5:GFP and the programmed cell death (PCD) caused by TMV P5. The latter was also observed with its ortholog, P6, encoded by CTV and another ER-localized protein, P34, encoded by LIYV RNA1. It is worth mentioning that LIYV P34 is an RNA-binding protein that is critical for LIYV RNA2 replication, however, the previous yeast two-hybrid assay showed no direct interaction of P5 and P34. Whether these two proteins could interact in vivo, their cooperative or separate functions on the ER is yet unclear [8,9]. Meanwhile, we have carefully quantified P5 expression at different levels. The P5 translated from LIYV infection was much less than that from the TMV vector (Figure S3), thus indicating that LIYV has evolved to regulate an adequate protein expression level to avoid damaging the cells, which may be necessary to promote virus persistence and spread. In previous studies, several ER-associated membrane proteins of plant-infecting viruses that induce ER stress have also been reported to regulate UPR-associated gene expression [35,36,41,42,43]. The UPR is a stress response conserved in eukaryotic organisms and activated to manage the accumulation of unfolded and misfolded proteins in the ER, to prolong cell viability, or under severe or chronic ER stress to initiate reactions leading to PCD [44,45]. Many mammalian RNA viruses manipulate host UPR signaling pathways to promote viral protein synthesis and persistence in infected cells [46,47]. In our study, we also observed the upregulation of several UPR indicator genes in TMV-P5, P34 and CTV P6 infiltrated areas, as a preliminary result, indicating a stress response from the host plants.

LIYV P9 shows no conserved sequences or functional motifs in existing databases, but it is important to note that P9 is conserved among all *Criniviruses*. Previous studies showed that it self-interacted and *Crinivirus* P9 orthologs, while showing no significant amino acid similarity, do show predicted similar secondary structure [17]. P9X showed the lowest viral titer of all constructs examined here, indicating it has a critical role for efficient LIYV infection of plants.

CTV, a *Closterovirus*, encodes three nonstructural proteins (P33, P18 and P13) that have been shown to be required only for systemic infection of some citrus host plants, but not others [48,49]. Whether P5 and P9 are similar in that regard is not yet known, but clearly both P5X and P9X gave less robust infections than the WT LIYV, and in the case of P5, this was seen for two different host plants, *N. benthamiana* and lettuce. Unfortunately, we could not obtain the P9X-infected lettuce plants, very likely due to the low quantities of virions we could extract from the P9X-agroinfected *N. benthamiana* plants to use for the membrane feeding assay. Thus, both of these small proteins, while not essential, provide functions for efficient LIYV infection.

## Figures and Tables

**Table 1:** Transmembrane (TM) domain prediction of the LIYV P5 amino acid sequence.

Algorithm	No. of TM Segment(s)	Starting-Ending aa ^a^
SOSUI	1	5–26
DAS	1	5–27
TMHMM	1	4–26
CCTOP	1	4–25
Phobius	1	6–24
TOPCONS	1	5–25

^a^ aa, amino acid.

## Supplementary Materials

The following are available online at http://www.mdpi.com/1999-4915/10/9/459/s1, Table S1: Primer sequences used to quantify the transcript levels of the stress-related *Nicotiana benthamiana* genes, Figure S1: The impact of *P5* and *P9* gene deletions (P5Δ, P9Δ) on LIYV infection in *Nicotiana benthamiana* plants, Figure S2: The virus accumulation level of LIYV P5 and P9 mutants visualized under UV light, Figure S3: Examination of P5-expressing TMV vectors.
