# Supplementary material for: Two Crinivirus-Conserved Small Proteins, P5 and P9, Are Indispensable for Efficient Lettuce infectious yellows virus Infectivity in Plants
Source: Viruses. 2018 Aug 28;10(9):459. doi: 10.3390/v10090459 (PMC6163742; doi:10.3390/v10090459)
Supplement: Supplementary file 1 [file viruses-10-00459-s001.pdf]

viruses

MDPI

Supplementary Materials

Table S1. Primer sequences used to quantify the transcript levels of the stress-related *Nicotiana benthamiana* genes.

Gene amplified	Primer sequences
BLP-4	Forward: 5'-AGCTTTGAGCAGTCAACACCAAGT-3'<br>Reverse: 5'-AAAACGTGCCCGAGTAAGTGGTTC-3'
bZIP60	Forward: 5'-CCTGCTTTGGTTCATGGGCATCAT-3'<br>Reverse: 5'-AGAAGACCGTGGTTTCTGCTTCGT-3'
PDI	Forward: 5'-TCCAAAGGGATCACTGGAGCCAAA-3'<br>Reverse: 5'-TCTGGAGATAGCACCACAACGCTT-3'
CRT	Forward: 5'-TGATTGGGACCTTCTCCCACCAAA-3'<br>Reverse: 5'-TCTGGCTTCTTGGCATCAGGATCA-3'
CAM	Forward: 5'-ATCTGCTAACGAGCTGAGGCATGT-3'<br>Reverse: 5'-TGACCATCACCATCCAAGTCTGCT-3'
SKP1	Forward: 5'-TGACATGCCAGACAGTTGCAGACA-3'<br>Reverse: 5'-AGGCATTCTCCCTCCTGACTTCTT-3'
18S rRNA	Forward: 5'-ATGGCCGTTCTTAGTTGGTGGAGC-3'<br>Reverse: 5'-AGTTAGCAGGCTGAGGTCTCGAAC-3'

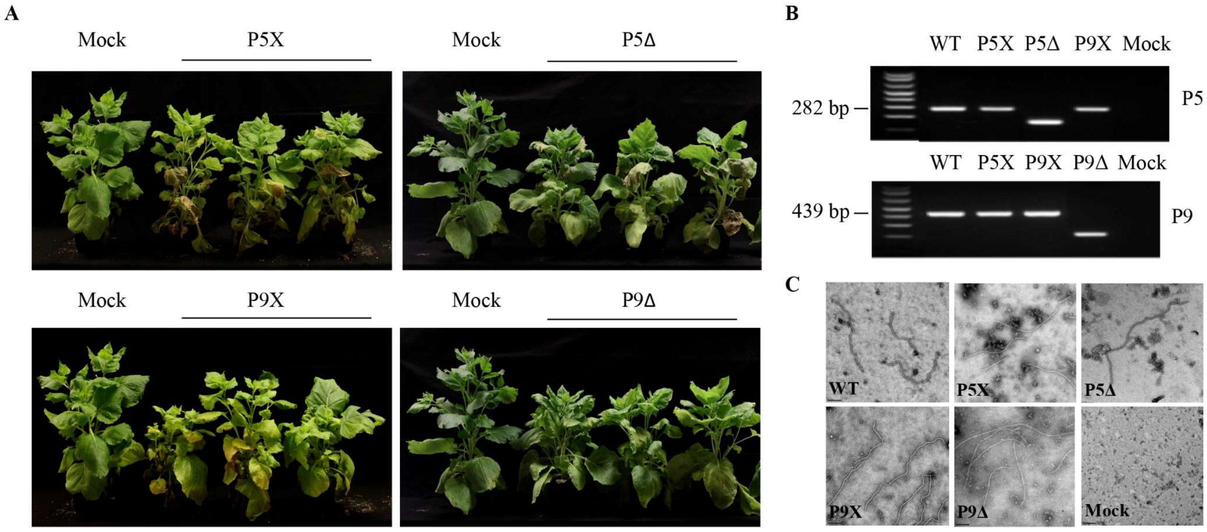

Figure S1. The impact of P5 and P9 gene deletions (P5Δ, P9Δ) on LIYV infection in *Nicotiana benthamiana* plants. (**A**) Phenotypes of LIYV P5X, P5Δ, P9X and P9Δ infected *N. benthamiana* plants photographed at 4 weeks post inoculation (wpi). (**B**) Mutations were examined by RT-PCR using total RNAs extracted from upper non-inoculated leaves of the infected plants. LIYV P5 and P9 primer sets were used to amplify the sequence flanking the P5 and P9 ORFs. (**C**) Electron microscopy of partially purified virions from the upper non-inoculated leaves of LIYV WT and mutants infected *N. benthamiana* leaves. Mock indicates buffer-inoculated control.

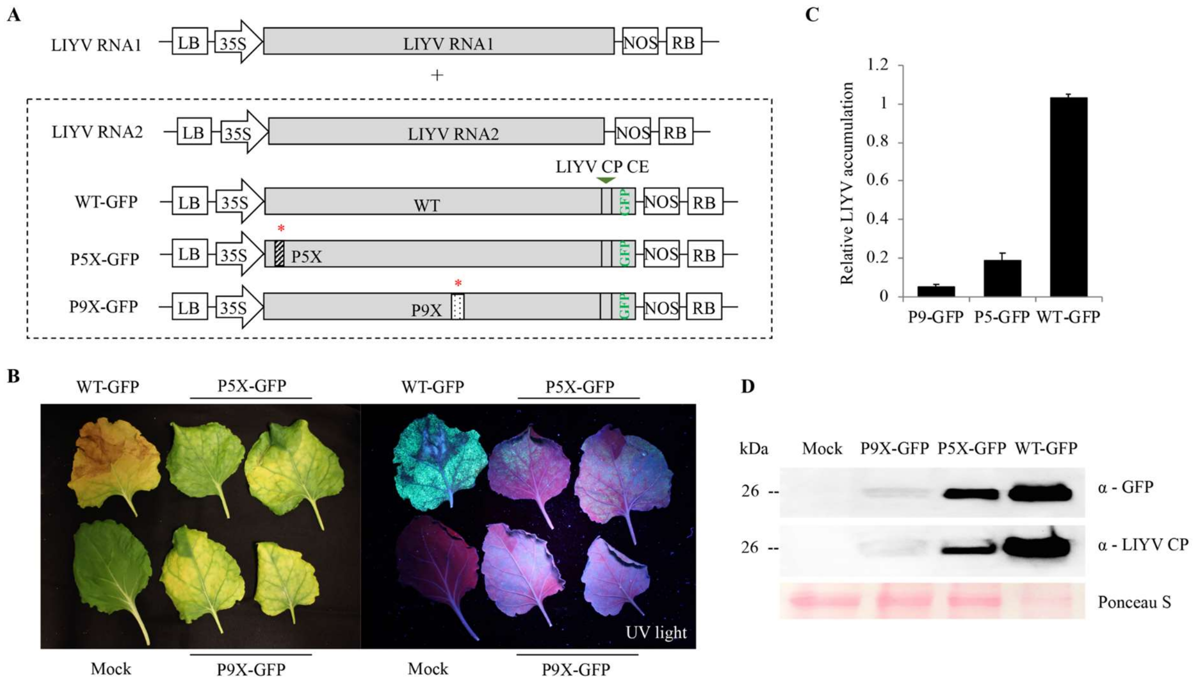

Figure S2. The virus accumulation level of LIYV P5 and P9 mutants visualized under UV light. (**A**) Schematic diagram of the genome organization of the GFP-tagged LIYV WT (WT-GFP), P5X (P5X-GFP) and P9X (P9X-GFP) cDNA infectious clones. A GFP open reading frame (ORF) controlled by a 150 bp duplicated LIYV CP controller element (CE) was inserted between P26 ORF and 3’-nontranslated region (NTR) of LIYV RNA2 [28]. (**B**) LIYV symptoms in upper non-inoculated leaves of LIYV WT-GFP, P5X-GFP and P9X-GFP infected *Nicotiana benthamiana* plants at 4 wpi (left) and GFP fluorescence visualized under UV light (right). Mock indicates buffer-inoculated control. (**C**) Quantification of LIYV RNA1 accumulation in LIYV WT-GFP, P5X-GFP and P9X-GFP infected *N. benthamiana* plants by RT-qPCR. The PP2A transcript level of lettuces was used as an internal control. Error bars denote standard errors from at least three biological replicates. (**D**) Immunoblot analysis of the GFP and CP expression in upper non-inoculated leaves of LIYV-infected plants using GFP and LIYV CP specific antibodies. The Ponceau S stained rubisco large subunit serves as a loading control.

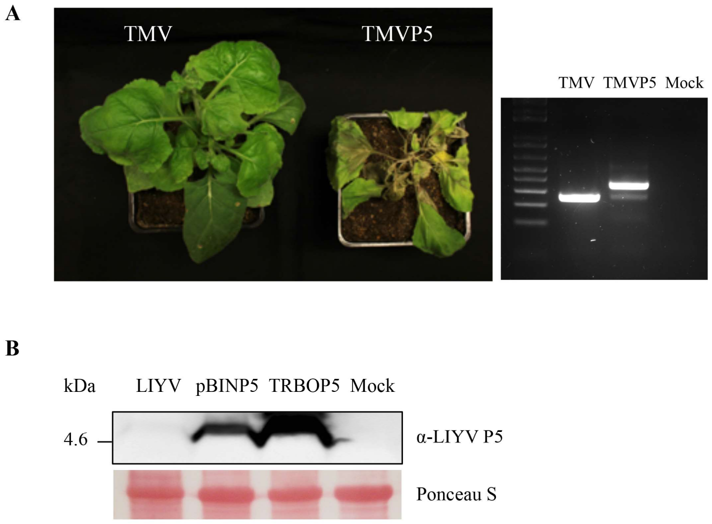

Figure S3. Examination of P5-expressing TMV vectors. (**A**) Left: Phenotypes of *Nicotiana benthamiana* plants inoculated with TMV and TMV expressing P5; Right: Systemic infection and P5 insertion were confirmed by RT-PCR with TMV specific primers flanking the insertion site. (**B**) Immunoblot analysis of P5 expressed from upper leaves of LIYV systemically infected *N. benthamiana* plants (LIYV), from a binary vector (pBINP5) and a TMV vector (TRBOP5) in agroinfiltrated *N. benthamiana* leaves.
